# Assessing the causal relationship between 731 immunophenotypes and the risk of lung cancer: a bidirectional mendelian randomization study

**DOI:** 10.1186/s12885-024-12014-1

**Published:** 2024-02-26

**Authors:** Ming Xu, Chengkai Li, Liyan Xiang, Siyue Chen, Lin Chen, Gongxia Ling, Yanqing Hu, Lan Yang, Xiang Yuan, Xiaodong Xia, Hailin Zhang

**Affiliations:** 1 The Second Affiliated Hospital, Yuying Children’s Hospital, Wenzhou Medical University, 109 West Xueyuan Road, Lucheng District, Zhejiang 325007 Wenzhou, PR China; 2 Department of Children’s Respiration Disease, The Second Affiliated Hospital and Yuying Children’s Hospital, Wenzhou Medical University, 109 West Xueyuan Road, Lucheng District, 325027 Wenzhou, Zhejiang PR China

**Keywords:** Lung cancer, Immunity, Causal inference, Mendelian randomization

## Abstract

**Background:**

Previous studies have observed a link between immunophenotypes and lung cancer, both of which are closely associated with genetic factors. However, the causal relationship between them remains unclear.

**Methods:**

Bidirectional Mendelian randomization (MR) was performed on publicly available genome-wide association study (GWAS) summary statistics to analyze the causal relationships between 731 immunophenotypes and lung cancer. Sensitivity analyses were conducted to verify the robustness, heterogeneity, and potential horizontal pleiotropy of our findings.

**Results:**

Following Bonferroni adjustment, CD14^−^ CD16^+^ monocyte (OR = 0.930, 95%CI 0.900–0.960, *P* = 8.648 × 10^− 6^, *P*_Bonferroni_ = 0.006) and CD27 on CD24^+^ CD27^+^ B cells (OR = 1.036, 95%CI 1.020–1.053, *P* = 1.595 × 10 − 5, *P*_Bonferroni_ = 0.012) were identified as having a causal role in lung cancer via the inverse variance weighted (IVW) method. At a more relaxed threshold, CD27 on IgD^+^ CD24^+^ B cell (OR = 1.035, 95%CI 1.017–1.053, *P* = 8.666 × 10^− 5^, *P*_Bonferroni_ = 0.063) and CD27 on switched memory B cell (OR = 1.037, 95%CI 1.018–1.056, *P* = 1.154 × 10^− 4^, *P*_Bonferroni_ = 0.084) were further identified. No statistically significant effects of lung cancer on immunophenotypes were found.

**Conclusions:**

The elevated level of CD14^−^ CD16^+^ monocytes was a protective factor against lung cancer. Conversely, CD27 on CD24^+^ CD27^+^ B cell was a risk factor. CD27 on class-switched memory B cells and IgD^+^ CD24^+^ B cells were potential risk factors for lung cancer. This research enhanced our comprehension of the interplay between immune responses and lung cancer risk. Additionally, these findings offer valuable perspectives for the development of immunologically oriented therapeutic strategies.

**Supplementary Information:**

The online version contains supplementary material available at 10.1186/s12885-024-12014-1.

## Background

Lung cancer represents a significant global health issue. It ranks as one of the most common types of cancer and is a major cause of cancer-related death, which is responsible for approximately 2 million new cases and 1.76 million deaths, annually [1]. This disease is primarily divided into two categories: small-cell lung cancer (SCLC) and non-small-cell lung cancer (NSCLC), the latter encompassing subtypes such as lung squamous cell cancer (LUSC) and lung adenocarcinoma (LUAD) [2, 3]. The occurrence of lung cancer is on the rise, particularly in developing countries. This increase is attributed to factors like wider availability of tobacco products and the environmental effects of industrialization [4]. Smoking remains the primary risk factor, responsible for a significant percentage of lung cancer cases in both males and females [5]. Additionally, exposure to ambient particulate matter pollution significantly contributes to lung cancer deaths [6, 7]. Beyond environmental causes, genetic mutations are also key in the development of lung cancer [8]. Genome-wide association studies (GWAS) have been vital in identifying genetic factors that increase lung cancer risk, especially those linked to smoking habits and DNA repair processes [9].

The interplay between immune cells and lung cancer, especially NSCLC, represents a complex and dynamic field of research. Within the tumor microenvironment of NSCLC, immune responses are intricately influenced by genomic aberrations, alterations in chromatin architecture, and the activity of non-coding RNAs [10, 11]. These elements are pivotal in tumor initiation, progression, and determining the immunogenic profile of the cancer [12, 13]. Chronic inflammation is an important risk factor in cancer progression, which promote the tumor growth [14–16]. The existence of persistent inflammation promotes tumor progression through various mechanisms such as immune system evasion, angiogenesis, and the facilitation of metastatic spread [17–19]. Recent studies have focused on the complex interactions between immune cells and lung cancer cells [20–23]. This complexity is evident by the formation of heterotypic cell-in-cell structures (CICs), highlighting the interplay in the tumor environment [17, 24].

The response of both innate and adaptive immune cells to different anti-cancer therapies, including chemotherapy, targeted therapy, and immune checkpoint blockade (ICB), are still not fully understood [25]. In lung cancer, a pro-tumorigenic immune response is often identified by enhanced immune checkpoint activity and the prevalence of immunosuppressive cells, combined with a concurrent reduction in anti-tumor immune mechanisms [26]. This immunological balance is associated with tumor progression and poor clinical outcomes [27]. Monocytes have complicated functions in the lung cancer microenvironment: they can promote or inhibit tumor growth, primarily differentiating into macrophages [28]. Notably, classical and intermediate monocytes are more prevalent in lung cancer patients, indicating their potential role in the pro-cancer immune response [29]. However, the exact causal relationship between immune cells and lung cancer remained unclear.

**Fig. 1:**
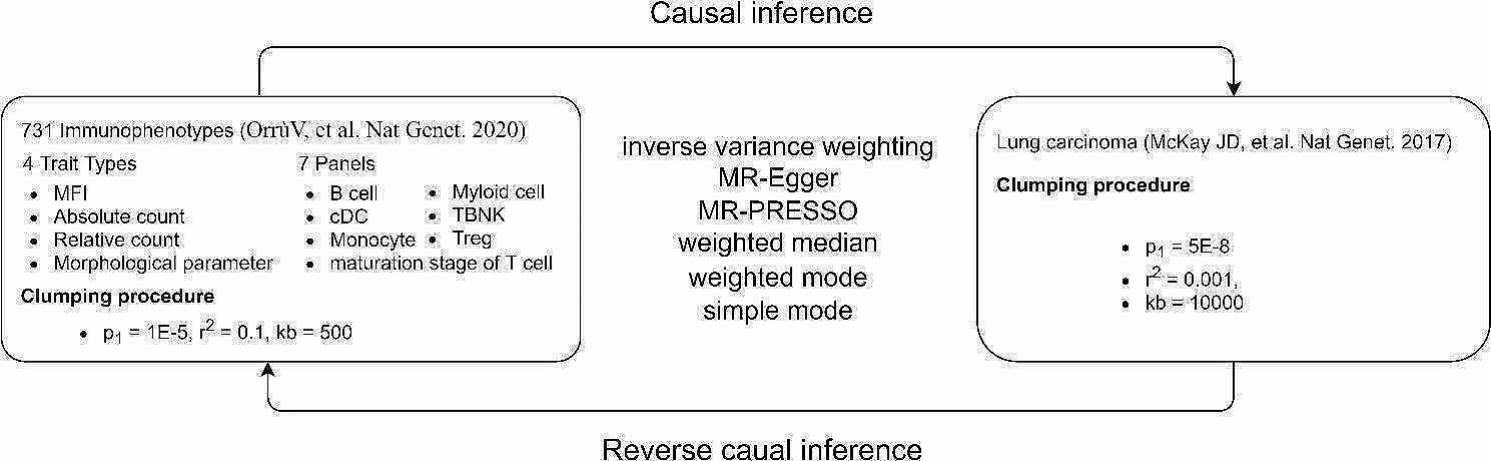
Workflow of the entire research

Advances in large-scale GWAS and Mendelian randomization (MR) methods have made it possible to assess causal associations between immune profiles and disease outcomes [30, 31]. Numerous studies have demonstrated the effectiveness of MR studies in exploring causal associations in lung cancer by avoiding confounders and reverse causal associations [32–34]. Therefore, our study used MR analysis to explore bidirectional causal associations between 731 immunophenotypes and lung cancer risk. The workflow of our study is shown in Fig. 1.

## Methods

### Study design

Our study was based on MR, which employs genetic variation as an instrumental variable (IV) to establish causality between exposure and outcome, mitigating bias arising from the confounding reverse causality issue. Selection of IVs follows three key rules in MR analysis: (1) direct association of genetic variation with exposure; (2) absence of association between genetic variation and potential confounders between exposure and outcome; and (3) genetic variation has no influence on outcome through pathways other than exposure.

A bi-directional two-sample MR analysis was conducted to evaluate the casual relationship between 731 immune phenotypes (7 immune panels) and lung cancer, based on summary-level datasets of large-scale GWAS studies. All GWAS studies included in this paper were ethically approved by their institutions.

### GWAS data sources

GWAS data for immune phenotypes were extracted from GWAS Catalog (accession numbers from GCST0001391 to GCST0002121) [30]. The study encompassed 731 immunophenotypes, including 118 absolute cell counts (AC), 389 median fluorescence intensities (MFI), 32 morphological parameters (MP), and 192 relative cell counts (RC). With data from 3,757 European individuals, the investigation identified 122 significant independent association signals at 70 loci, revealing 53 novel loci and clarified the regulatory mechanisms of 459 cellular signatures. Genotyping employed four Illumina arrays and approximately 22 million SNPs, with imputation based on a Sardinian sequence-based reference panel. Covariate-adjusted association analyses were performed, considering sex, age, and age^2^.

GWAS summary statistics for lung cancer were download from a large-scale GWAS Meta-analysis (accession number GCST004748) of European ancestry (N_case_ = 29,266, N_control_ = 56,450), with approximately 10.4 million SNPs analyzed after quality control and imputation [35]. This study identified 18 susceptibility loci under the level of genome-wide significance (*P* < 5 × 10^− 8^), 10 of which were novelly discovered.

### Selection of instrumental variables (IVs)

In this study, we extracted independent and significant SNPs for each immune trait with a significance threshold of 1 × 10^− 5^. This threshold is less stringent than the commonly used 5 × 10^− 8^, as suggested by previous studies that smaller sample size in immunophenotypes do not require such stringent p-value correction thresholds [30, 36–38]. Linkage disequilibrium (LD) analysis was performed with a r^2^ threshold < 0.1 within a 500 kb distance. For lung cancer, the significance level was adjusted to 5 × 10^− 8^, and LD analysis was performed with a r^2^ threshold < 0.001 within a 10,000 kb distance. When possible, instrumental Single-Nucleotide Polymorphisms (SNPs) for the exposure absent in the outcome datasets were proxied using SNPs in high linkage disequilibrium (r^2^ > 0.8). All SNPs were harmonized between the exposure and the outcome by alleles to ensure the alignment of effect. The F-statistic were calculated for each IV through this formula to avoid weak instrumental bias: F = R^2^×(*N* − 2)/(1 − R^2^); R^2^ = 2×EAF×(1 − EAF)×β^2^ [39]. In this formula, R^2^ refers to the cumulative explained variance of the selected IVs, and EAF refers to the effect allele frequency, β refers to the estimated effect of SNP, and N refers to the sample size of the GWAS. SNPs with F-statistic values greater than 10 were considered strong instrumental variables and were retained in subsequent analyses. Confounders were detected using Phenoscanner V2, SNPs associated with both exposure and outcomes were removed from the study [40]. The GWAS data on exposures and outcomes used in this paper are from different studies, so there is no population overlap.

### Statistical analysis

Multiple MR methods were employed to assess the causal relationship between 731 immunophenotypes and lung cancer, including inverse variance weighting (IVW), weighted median, MR-Egger, simple mode, and weighted mode. The IVW method is employed as our primary MR analysis, and it applies a meta-analysis method to integrate the Wald ratio of individual SNPs, which is assumed that instrumental variables (IVs) exclusively influence outcomes through specified exposure, rendering unbiased causal estimates in the absence of horizontal pleiotropy [41]. Therefore, the IVW method provides the most accurate assessment at the absence of horizontal pleiotropy [42]. To complement our analysis and identify potential biases arising from ineffective IVs and horizontal pleiotropy, we integrated the weighted median method and MR-Egger method [43]. However, these results might be inaccurate, as they may be susceptible to the impact of outlier genetic variants, especially MR-Egger method [44]. The weighted median method, while exhibiting a relatively small bias, is characterized by lower precision, particularly in cases where the percentage of IVs with horizontal pleiotropy is less than 50% [45].

Sensitivity analysis is done to assess potential heterogeneity and horizontal pleiotropy. Cochran’s Q test evaluated the heterogeneity of effect sizes for selected genetic IVs. Additionally, we applied the MR-Pleiotropy Residual Sum and Outlier (MR-PRESSO) method to identify and exclude outliers and moderate horizontal pleiotropy. The intercept derived from MR-Egger regression served to evaluate vertical pleiotropy [46]. Leave-one-out analysis was conducted to examine the impact of removing individual selected SNPs on the overall results [47]. Scatter plots were used to confirm the absence of outlier influence on the results. Additionally, funnel plots were employed to assess the robustness of correlation and the absence of heterogeneity. To address multiple testing, all p-values for MR analysis were corrected using the Bonferroni method. We employed 2 Bonferroni-corrected p-value thresholds set at 0.05 and 0.1. *P*_*Bonferroni*_ below 0.05 were considered statistically significant, whereas those between 0.05 and 0.1 categorized as potentially statistically significant. P-values for sensitivity analysis were uncorrected to avoid increasing the false-negative rate.

All analyses were performed in R 4.3.1 software (http://www.Rproject.org). Main MR analysis was performed using the “TwoSampleMR” package (version 0.5.7). MR-PRESSO was performed using the ‘MR-PRESSO’ package. Confounders were removed by calling “phenoscanner V2” (http://www.phenoscanner.medschl.cam.ac.uk/) in the ‘Mendelian Randomization’ package.

## Results

### Genetic instrumental variant selection

In this study, we identified a median count of 27 (range 2–1217) independent IVs associated with 731 immunophenotypes. These identified IVs accounted for an average of 0.178% of the phenotypic variance, with the range from 0.005 to 5.199% (Table S1 for details). While most SNPs are associated with only one immunophenotype, there are also SNPs that correlate with multiple immunophenotypes (detailed in Table S6). Moreover, all computed F-statistics were above 19.53. For lung cancer, 15 IVs (3 removed for correlating with immunophenotypes, 2 for association with smoking) were identified for further reverse-directional analytical investigations (Table S5).

### The causal role of immunophenotypes on lung cancer

**Fig. 2:**
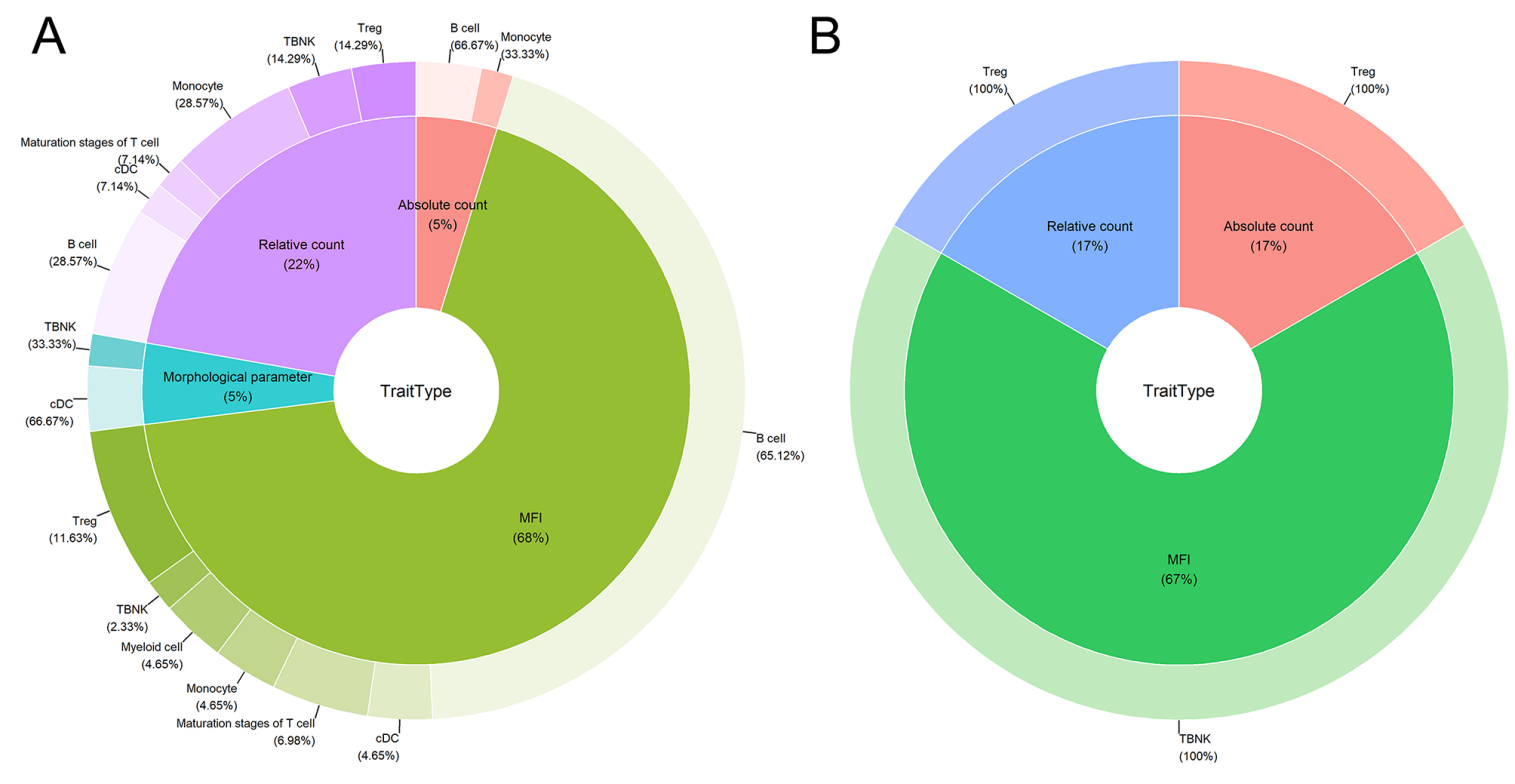
The distribution of immune cells exhibiting significance at a nominal significance level varies across distinct trait categories and diverse analytical panels. (**A**) The causal effects of immune cell profiles on the risk of lung cancer. (**B**) The causal role of lung cancer on immune cells

Prior to adjustment, a total of 63 immune phenotypes were identified as having a causal role. This includes the elevation of 40 immune cell types and the reduction of 23, which are implicated in the induction of lung cancer (Table S2A). The distribution of these 63 immune cell types spans several categories: B cells (34 types), monocytes (7 types), regulatory T cells (Tregs, 7 types), conventional dendritic cells (cDCs, 5 types), maturation stages of T cells (4 types), T, B, natural killer (TBNK) cells (4 types), and myeloid cells (2 types), as visualized in Fig. 2A.

After Bonferroni adjustment (*P*_*Bonferroni*_ < 0.05), only two results retained statistical significance. For the monocyte panel, the odds ratio (OR) of CD14^−^ CD16^+^ monocyte was estimated to be 0.930 (95%CI 0.900–0.960, *P* = 8.648 × 10^− 6^, *P*_Bonferroni_ = 0.006) using IVW method. Additional analytical methods yielded the following results: MR-Egger (OR = 0.917, 95% CI 0.864–0.973, *P* = 0.008), weighted Median (OR = 0.932, 95% CI 0.888–0.978, *P* = 0.004), simple mode (OR = 0.966, 95% CI 0.884–1.055, *P* = 0.447), weighted mode (OR = 0.932, 95% CI 0.888–0.978, *P* = 0.004), MR-PRESSO (OR = 0.930, 95% CI 0.903–0.957 *P* = 3.176 × 10^− 5^, global *P* = 0.765). In the B cells panel, CD27 on CD24^+^ CD27^+^ B cells held the OR of 1.036 (95%CI 1.020–1.053, *P* = 1.595 × 10^− 5^, *P*_Bonferroni_ = 0.012) by IVW method. Results from other methods are listed as follows: MR-Egger (OR = 1.051, 95% CI 1.018–1.085, *P* = 0.003), weighted Median (OR = 1.041, 95% CI 1.014–1.068, *P* = 0.003), simple mode (OR = 1.030, 95% CI 0.979–1.084, *P* = 0.250), weighted mode (OR = 1.029, 95% CI 0.998–1.061, *P* = 0.072), MR-PRESSO (OR = 1.036, 95% CI 1.021–1.052 *P* = 1.385 × 10^− 5^, global *P* = 0.810).

**Fig. 3:**
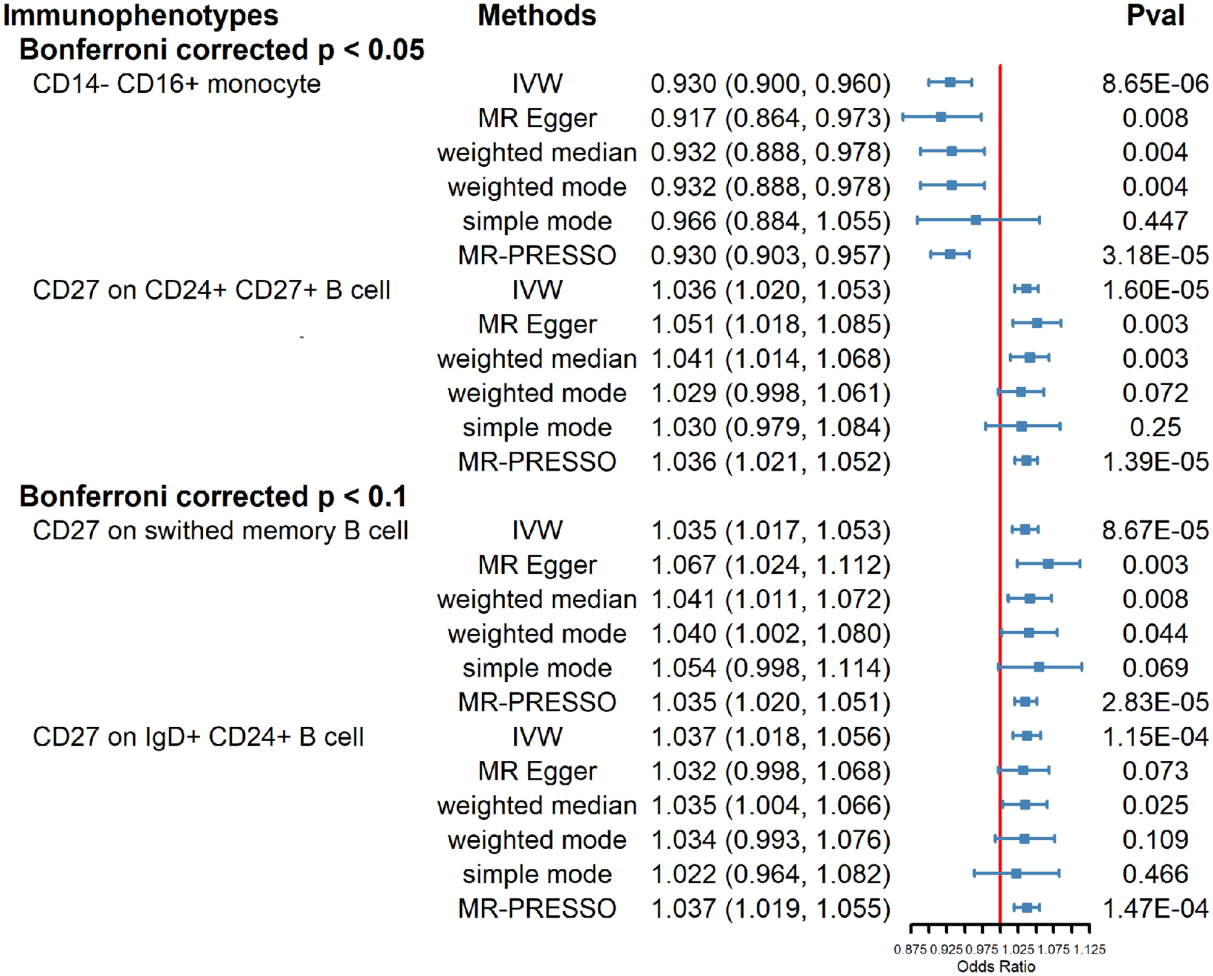
Causal associations between four identified immune phenotypes and the risk of lung cancer incidence with different MR methods

Upon relaxing the significance threshold to *P*_*Bonferroni*_ < 0.1, two additional significant findings were found within the B cells panel. The OR of CD27 on IgD^+^ CD24^+^ B cell was estimated to be 1.035 (95%CI 1.017–1.053, *P* = 8.666 × 10^− 5^, *P*_Bonferroni_ = 0.063) by IVW method. Results from other methods are listed as follows: MR-Egger (OR = 1.067, 95% CI 1.024–1.112, *P* = 0.003), weighted Median (OR = 1.041, 95% CI 1.011–1.072, *P* = 0.008), simple mode (OR = 1.054, 95% CI 0.998–1.114, *P* = 0.069), weighted mode (OR = 1.040, 95% CI 1.002–1.080, *P* = 0.044), MR-PRESSO (OR = 1.035, 95% CI 1.020–1.051 *P* = 2.831 × 10^− 5^, global *P* = 0.929). Similarly, CD27 on switched memory B cell presented an OR of 1.037 (95%CI 1.018–1.056, *P* = 1.154 × 10^− 4^, *P*_Bonferroni_ = 0.084) by IVW method. Other methods indicated the following: MR-Egger (OR = 1.032, 95% CI 0.998–1.068, *P* = 0.073), weighted Median (OR = 1.035, 95% CI 1.004–1.066, *P* = 0.025), simple mode (OR = 1.022, 95% CI 0.964–1.082, *P* = 0.466), weighted mode (OR = 1.034, 95% CI 0.993–1.076, *P* = 0.109), MR-PRESSO (OR = 1.037, 95% CI 1.019–1.055 *P* = 1.470 × 10^− 4^, global *P* = 0.681). The aforementioned results were visualized in Fig. 3 (Table S3 for detail).

The intercept analysis conducted through MR-Egger, alongside the global test implemented via MR-PRESSO, conclusively eliminated horizontal pleiotropy as a potential confounder in our findings (Table S4). Further validation was provided by the stability observed in the scatter plots, funnel plots and leave-one-out analysis, which proved the robustness of the results (Fig S1-3).

### Exploration of the causal effect of lung cancer on immunophenotypes

Before adjustment, lung cancer was observed to induce increase in 6 immunophenotypes (Table S2B). These immunophenotypic changes included: TBNK cells (4 types) and Tregs (2 types), as presented in Fig. 2B. However, after Bonferroni adjustment, none of these p values remained statistically significant.

## Discussion

Our research combines individual-level data from GWAS to explore how immune cells genetically contribute to the development and progression of lung cancer. This study presents evidence suggesting that immune cells may affect lung cancer risk through a comprehensive genetic analysis. SNPs were employed as instrumental variables in a bidirectional two-sample MR analysis. Our findings indicate a protective effect of CD14^−^ CD16^+^ monocytes against lung cancer. Moreover, we observed an association between CD27 on CD24^+^ CD27^+^ B cells and increased lung cancer risk. Additionally, the increase of CD27 on switched memory B cells and CD27 on IgD^+^ CD24^+^ B cells may be linked to lung cancer development.

We revealed that the elevated levels of CD14^−^ CD16^+^ monocytes, known as nonclassical monocytes or patrolling monocytes (PMo), have a protective role in lung cancer development. Monocytes is an important composition the innate immune system, which regulate cellular homeostasis and are classified into classical, intermediate, and nonclassical subtypes [48]. Unlike classical monocytes, which are implicated in tumorigenesis and cancer metastasis, nonclassical monocytes exhibit unique tumor interactions, particularly in the lung [49]. These monocytes are enriched in the lung’s microvasculature, where they reduce tumor metastasis, as evidenced in various mouse metastatic tumor models [50]. For instance, Nr4a1-deficient mice, which lack PMo, show increased lung metastasis. In contrast, transferring Nr4a1-proficient PMo into these mice impedes tumor invasion in the lung [51]. This subset of monocytes plays a crucial role in early interactions with metastasizing tumor cells, cleaning tumor material from the lung blood vessels, and assist in the recruitment and activation of natural killer cells. This activity is crucial for cancer immunosurveillance, and highlights their potential as targets for cancer immunotherapy [48]. Additional complexity with monocyte subsets is observed with Slan^+^ monocytes, identified by the 6-sulfo LacNAc (slan) antigen. These cells are a subset of non-classical monocytes in the human bloodstream with a significant role in cancer defense [52]. An exploratory study on SCLC patients treated with chemotherapy and immune checkpoint inhibitors indicated that low levels of slan^+^ non-classical monocytes correlate with poor survival across different histological types of lung cancer. This finding suggests that slan^+^ monocyte levels could help in predicting patient outcomes in lung cancer​ [53]. Additionally, Slan^+^ monocytes in lymphoma demonstrate potential roles in cancer immunity. They induced antibody-dependent cellular cytotoxicity (ADCC), particularly when interacting with therapeutic antibodies like Rituximab. This interaction leads to necrotic cell death through TNFα​ [54]. Slan^+^ monocytes also show remarkable plasticity, they can differentiate into distinct subsets of dendritic cells (DCs) and macrophages, especially in cancer tissues. These cells can acquire macrophage-like phenotypes and become efficient in rituximab-mediated antibody-dependent cellular phagocytosis (ADCP), and activate different FcγRs than those used by macrophages derived from CD14^+^ monocytes [55]. Furthermore, slan^+^ monocytes contribute to immune surveillance by producing pro-inflammatory cytokines and engaging in cross-talk with T cells and NK cells, amplifying immune responses against tumor cells [52]. However, the exact mechanisms and pathways involved in these processes in lung cancer remain not fully understood.

CD27 on CD24^+^ CD27^+^ B cell was identified to increase the risk of lung cancer in our research. CD24^hi^ CD27^+^ B cells are a subset of regulatory B cells (Bregs), which play a key role in immune regulation. A study examining the phenotypes of circulating Tregs and Bregs revealed a decreased frequency of Tregs and an increased frequency of Bregs (including CD24^hi^ CD27^+^ B cells) in patients with lung cancer. This finding suggest that lung cancer cells might directly interact with these cell types, so they may play a significant role in tumor development [56]. Bregs are notable for their high production of interleukin-10 (IL-10), a cytokine involved in immune response moderation. These cells are also efficient in suppressing CD4^+^ T cell proliferation and IFN-γ/IL-17 expression [57]. CD4^+^ T cells were essential in both innate and antigen-specific immune responses [58, 59], and play a crucial role in mobilizing the immune system against cancer cells​ [60]. The efficacy of CD4^+^ T cells in the peripheral blood of lung cancer patients has been correlated with improved anti-tumor responses. In fact, patients with better responses to treatments exhibit significantly higher percentages of specific CD4^+^ T cell types [61]. IFN-γ is essential in activating cellular immunity and triggering anti-tumor responses. It plays a significant role in lung cancer immunotherapy due to its ability to halt cell growth, promote cell death, and inhibit cell proliferation. IFN-γ also helps to slow down tumor growth by blocking blood vessel formation in tumors, promoting the death of regulatory T-cells, and boosting the activity of proinflammatory M1 macrophages [62]. High-dose IFN-γ treatment has even been observed to induce tumor regression [63]. It’s suggested that CD24^hi^ CD27^+^ B cells might promote lung cancer development by suppressing these anti-cancer factors.

Our analysis identified the expression of CD27 on class-switched memory B cells and IgD^+^ CD24^+^ B cells as potential risk factors for lung cancer, using a Bonferroni-adjusted significance threshold of *P*_*Bonferroni*_ < 0.1. However, little research was done to explore the potential function of these two immunophenotypes. CD27^+^ and IgD^−^ class-switched memory B cells (Bmems) constitute a greater proportion of the B cell population in pulmonary tissues compared to peripheral blood. Thus, how the accumulation of this immune phenotype in the lungs is associated with lung cancer development is currently unclear [64]. Additionally, the IgD^+^ CD27^+^ B cell phenotype is a subtype of unswitched memory B cells. CD24 is a molecule known for its roles in cell adhesion and signaling [65]. However, there is also a notable gap in the research concerning the direct impact of CD27 expression on IgD^+^ CD24^+^ B cells in the context of lung cancer development.

Interestingly, we found that while most SNPs are associated with only one immunophenotype, there are indeed SNPs that correlate with multiple immunophenotypes. Such overlap seems logical and may be explained by shared regulatory elements such as promoters or enhancers, or involvement in post-transcriptional modification processes among different immunophenotypes. The immunophenotypes we identified to have causal relationship on lung cancer have undergone rigorous sensitivity analyses, which significantly reduces concerns regarding horizontal pleiotropy.

Taken altogether, our study contributes to the understanding of lung cancer by highlighting the causal role of immune cells in its development. This finding is crucial for clinical decisions regarding disease prognosis and treatment. However, the pathogenesis of lung cancer involves complex interactions among various types of immune cells, and a single treatment approach is often insufficient. This highlights the need for further research into the interactions between innate and adaptive immune cells in lung cancer.

Our study did have limitations. The primary constraint is the reliance on GWAS summary datasets for immune traits and lung cancer, although they were the largest available. However, the differences in sample size, quality control methods, and ethnic composition may cause potential errors. Secondly, though rigorous sensitivity analyses reduce the concern for pleiotropy, a multivariable mendelian randomization (MVMR) analysis would be conducted to further explore the complex relationships between immunophenotypes and lung cancer ideally. However, due to the complexities (including computational demands, statistical power, the effectiveness of instrumental variables, and the challenges of clinically interpreting these complex results) from the 731 exposures in our study, using MVMR is currently not feasible. We hope that advances in methodology will enhance our understanding in this area in the future. Moreover, as these datasets were drawn from different studies, it is essential to interpret our findings with caution, despite our rigorous selection of IVs and extensive sensitivity analyses to mitigate potential confounding factors. Another limitation is the use of summary-level datasets, which precluded individual-level analysis and consequently, the inability to conduct population stratification studies based on variables like sex and age. Furthermore, while we applied the Bonferroni correction for multiple testing, which is the most stringent method, the selection of SNPs under a relatively relaxed threshold (1 × 10^− 5^) due to limited sample sizes may lead to some false positives.

In conclusion, our research offers new perspectives on the immunology of lung cancer onset. However, further experimental studies are needed to further understand the mechanisms linking immune traits with lung cancer. This could lead to more effective treatment strategies.

## Conclusions

The elevated level of CD14^−^ CD16^+^ monocytes was a protective factor against lung cancer. Conversely, CD27 on CD24^+^ CD27^+^ B cell was a risk factor. CD27 on class-switched memory B cells and IgD^+^ CD24^+^ B cells were potential risk factors for lung cancer.

Our study explored the causal influence of the immune response on lung cancer progression. This research enhanced our comprehension of the interplay between immune responses and lung cancer risk. Additionally, these findings offer valuable perspectives for the development of immunologically oriented therapeutic strategies.

### Electronic supplementary material

Below is the link to the electronic supplementary material.

Supplementary Material 1

Supplementary Material 2

Supplementary Material 3

Supplementary Material 4

Supplementary Material 5

## Data Availability

All GWAS data were available on GWAS Catalog (https://www.ebi.ac.uk/gwas/). Immunophenotypes: accession numbers from GCST0001391 to GCST0002121. Orr? V, Steri M, etal. Complex genetic signatures in immune cells underlie autoimmunity and inform therapy. Nat Genet. 2020 Oct;52(10):1036-1045. doi: 10.1038/s41588-020-0684-4IF: 30.8 Q1. Epub 2020 Sep 14. Erratum in: Nat Genet. 2020 Sep 18;: PMID: 32929287; PMCID: PMC8517961; lung cancer: accession number GCST004748. McKay JD, Hung RJ, etal. Large-scale association analysis identifies new lung cancer susceptibility loci and heterogeneity in genetic susceptibility across histological subtypes. Nat Genet. 2017 Jul;49(7):1126-1132. doi: 10.1038/ng.3892. Epub 2017 Jun 12. PMID: 28604730; PMCID: PMC5510465.

## Abbreviations

ADCC: Antibody-Dependent Cellular Cytotoxicity
ADCP: Antibody-Dependent Cellular Phagocytosis
Bmems: Memory B Cells
Bregs: Regulatory B Cells
CICs: Cell-in-Cell Structures
DCs: Dendritic Cells
GWAS: Genome-Wide Association Study
ICB: Immune Checkpoint Blockade
IVs: Instrumental Variables
IVW: Inverse Variance Weighted
LD: Linkage Disequilibrium
LUAD: Lung Adenocarcinoma
LUSC: Lung Squamous Cell Cancer
MFI: Median Fluorescence Intensities
MR: Mendelian Randomization
MR-PRESSO: MR-Pleiotropy Residual Sum and Outlier
NSCLC: Non-Small-Cell Lung Cancer
PMo: Patrolling Monocytes
RC: Relative Cell Counts
SCLC: Small-Cell Lung Cancer
slan: 6-sulfo LacNAc
SNPs: Single-Nucleotide Polymorphisms
