# Supplementary figures and images for: Assessing the causal relationship between 731 immunophenotypes and the risk of lung cancer: a bidirectional mendelian randomization study
Source: BMC Cancer. 2024 Feb 26;24:270. doi: 10.1186/s12885-024-12014-1 (PMC10898084; doi:10.1186/s12885-024-12014-1)

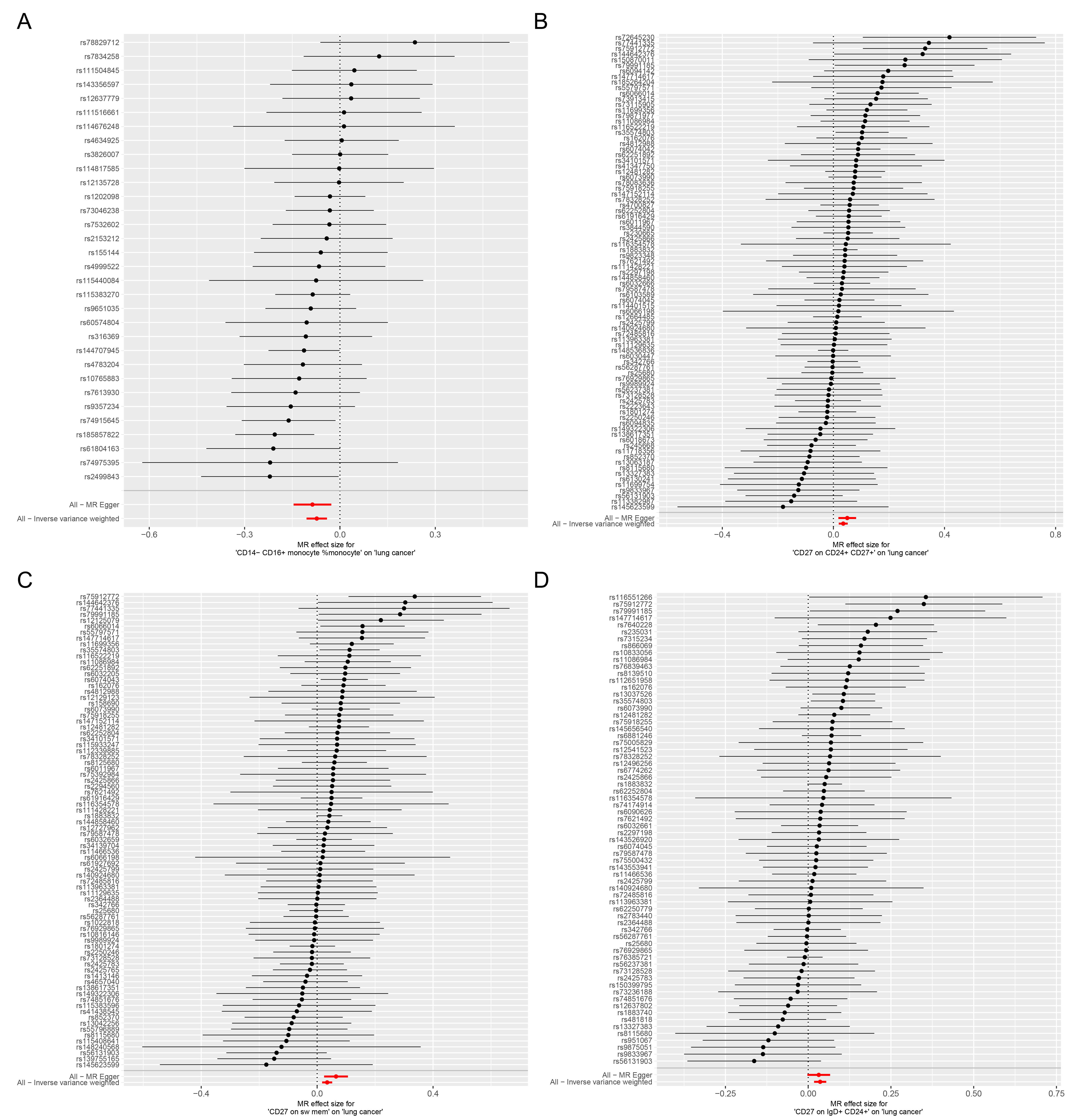

Supplement: Supplementary file 2 — Supplementary Material 2 [file 12885_2024_12014_MOESM2_ESM.png]

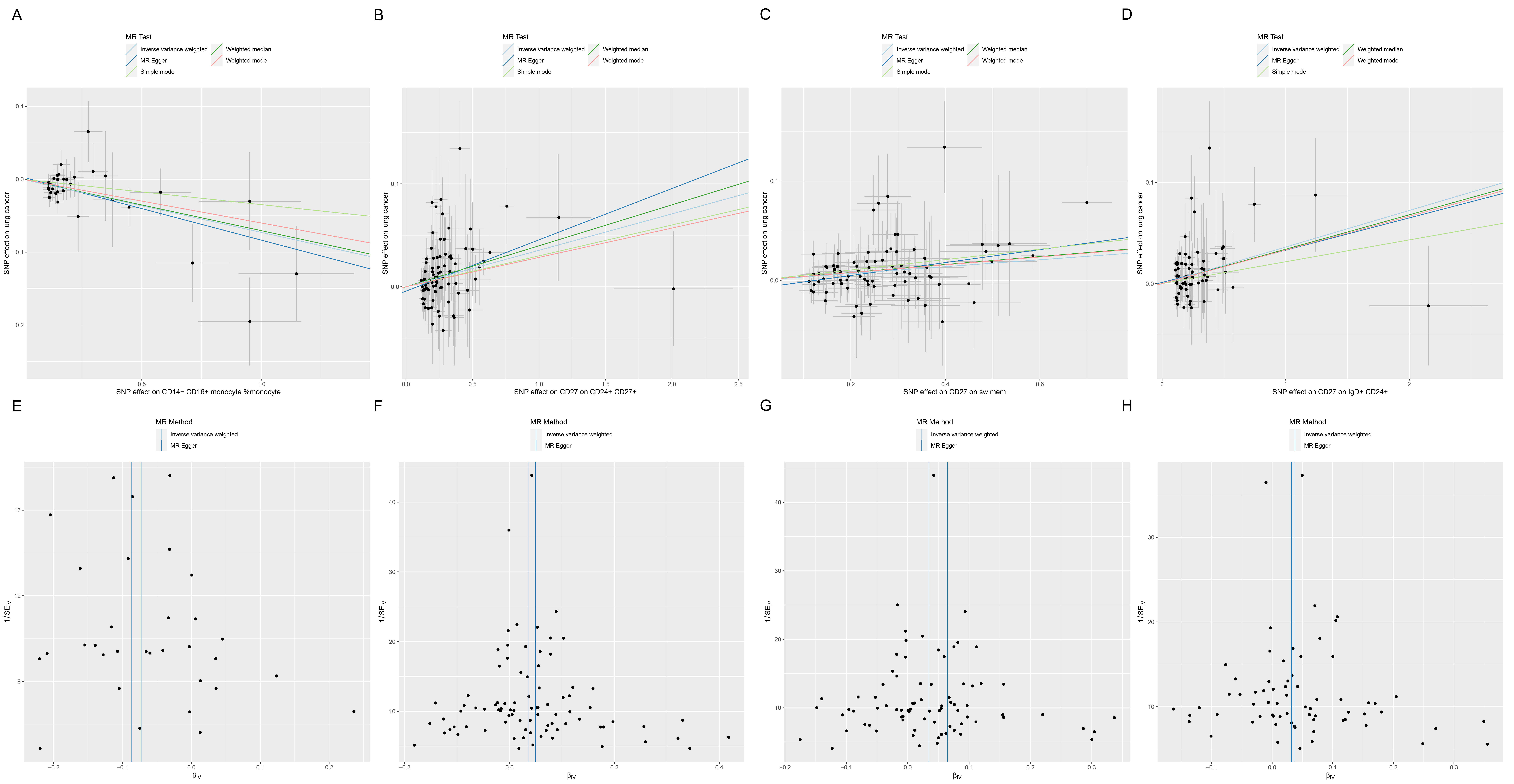

Supplement: Supplementary file 3 — Supplementary Material 3 [file 12885_2024_12014_MOESM3_ESM.png]

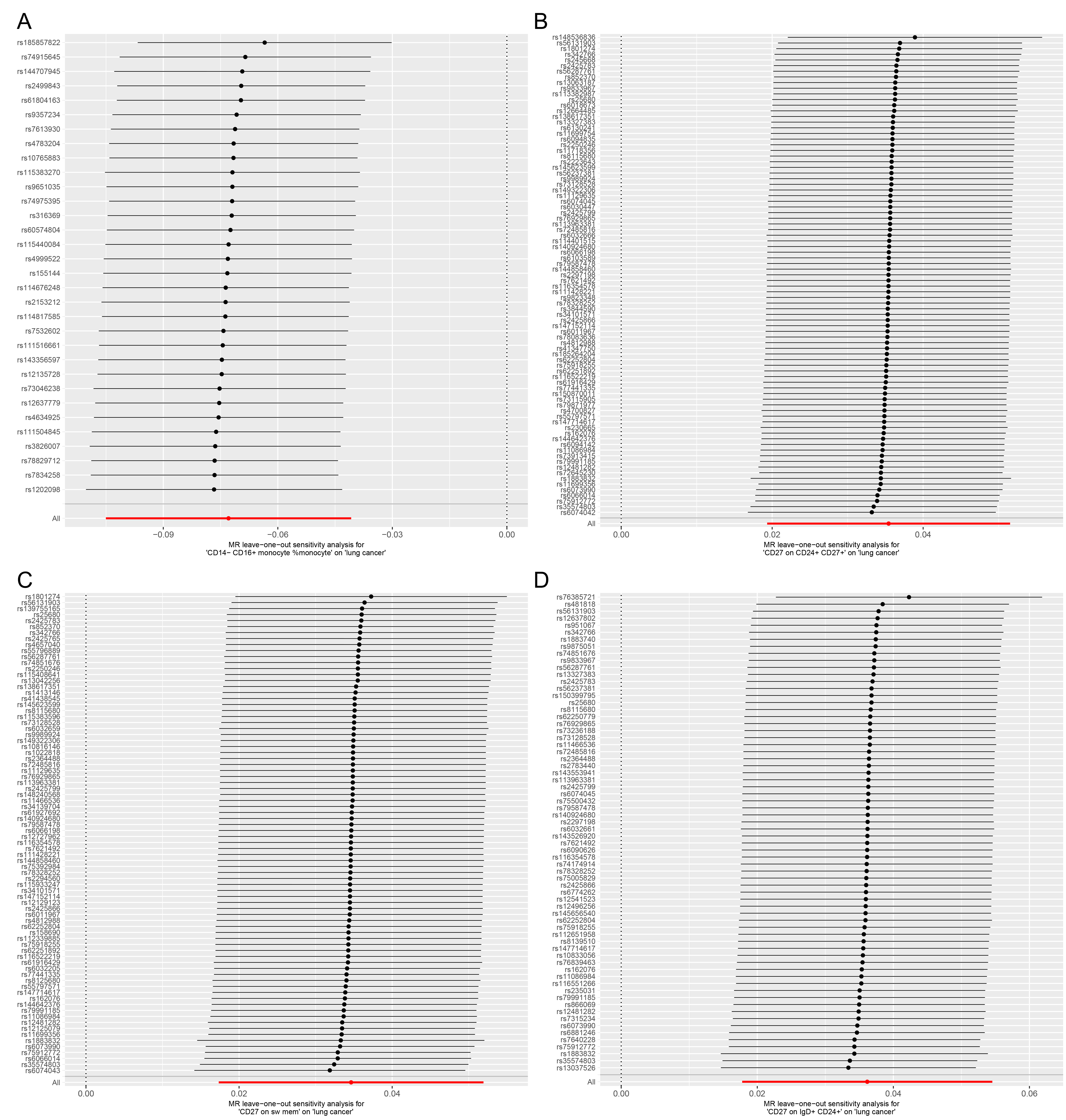

Supplement: Supplementary file 4 — Supplementary Material 4 [file 12885_2024_12014_MOESM4_ESM.png]
