# Supplementary material for: Assessing the causal relationship between 731 immunophenotypes and the risk of lung cancer: a bidirectional mendelian randomization study
Source: BMC Cancer. 2024 Feb 26;24:270. doi: 10.1186/s12885-024-12014-1 (PMC10898084; doi:10.1186/s12885-024-12014-1)
Supplement: Supplementary file 5 — Supplementary Material 5 [file 12885_2024_12014_MOESM5_ESM.docx]

**Supplementary figures**

**Figure S1:** Scatter plot and funnel plot

**Figure S2:** The forest plot

**Figure S3:** Leave-one-out plot of sensitivity analysis


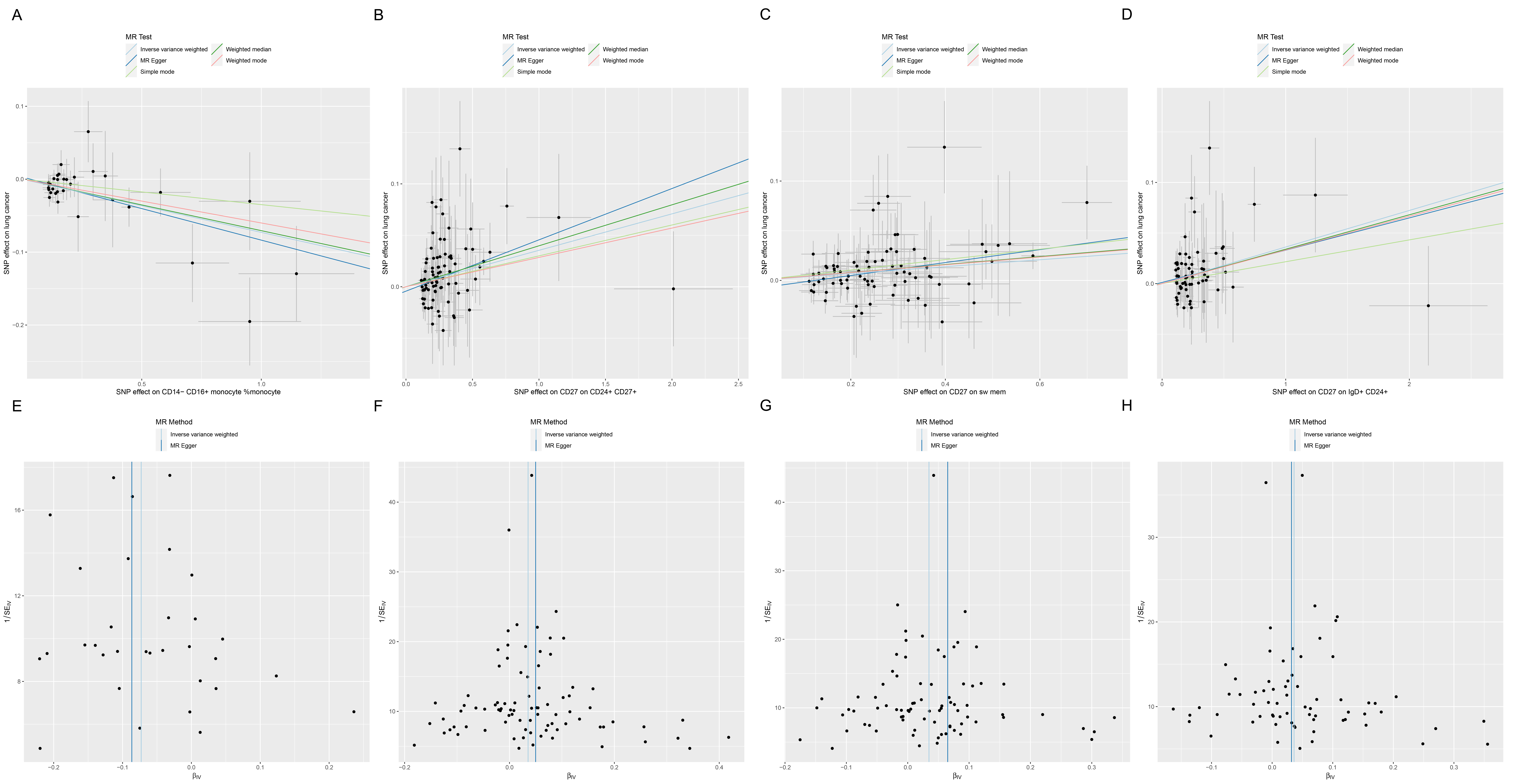
**Figure S1:** Scatter plot visualized the direction of the effects. The x-axis is the size of the SNP effect of immune cells, and the y-axis is the size of the SNP effect of the lung cancer. Different MR methodologies are distinguished by unique color coding (A: CD14^-^ CD16^+^ monocyte, B: CD27 on CD24^+^ CD27^+^ B cell, C: CD27 on switched memory B cell, D: CD27 on IgD^+^ CD24^+^ B cell). The funnel plot is utilized to evaluate publication bias and heterogeneity (E: CD14^-^ CD16^+^ monocyte, F: CD27 on CD24^+^ CD27^+^ B cell, G: CD27 on switched memory B cell, H: CD27 on IgD^+^ CD24^+^ B cell).


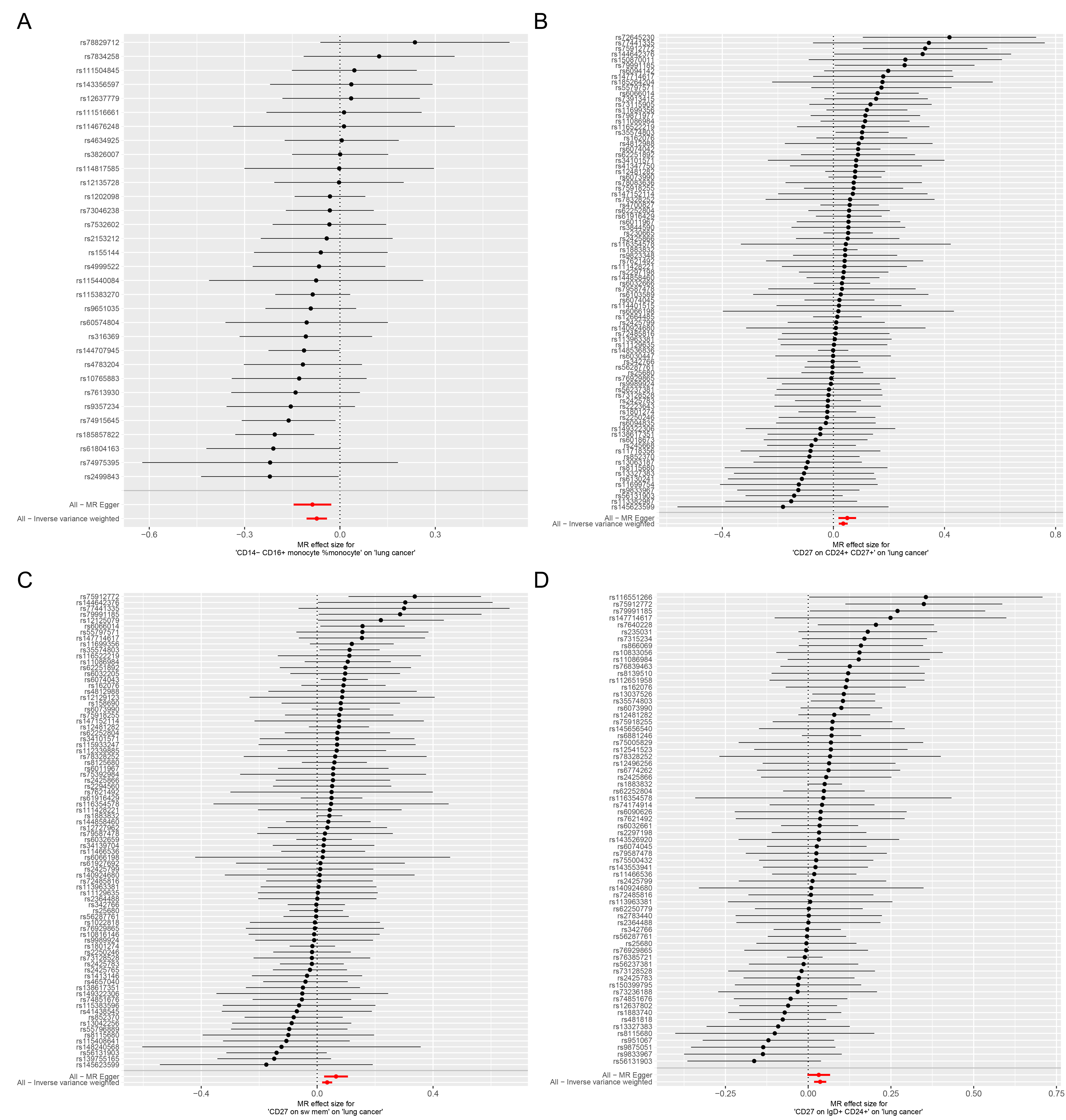
**Figure S2:** The forest plot illustrates the causal effect of IVs on lung cancer. Each dot represents the estimated effect size of a specific genetic variant, with horizontal lines denoting the 95% CI. The overall estimates are highlighted with red dots and lines (A: CD14^-^ CD16^+^ monocyte, B: CD27 on CD24^+^ CD27^+^ B cell, C: CD27 on switched memory B cell, D: CD27 on IgD^+^ CD24^+^ B cell).


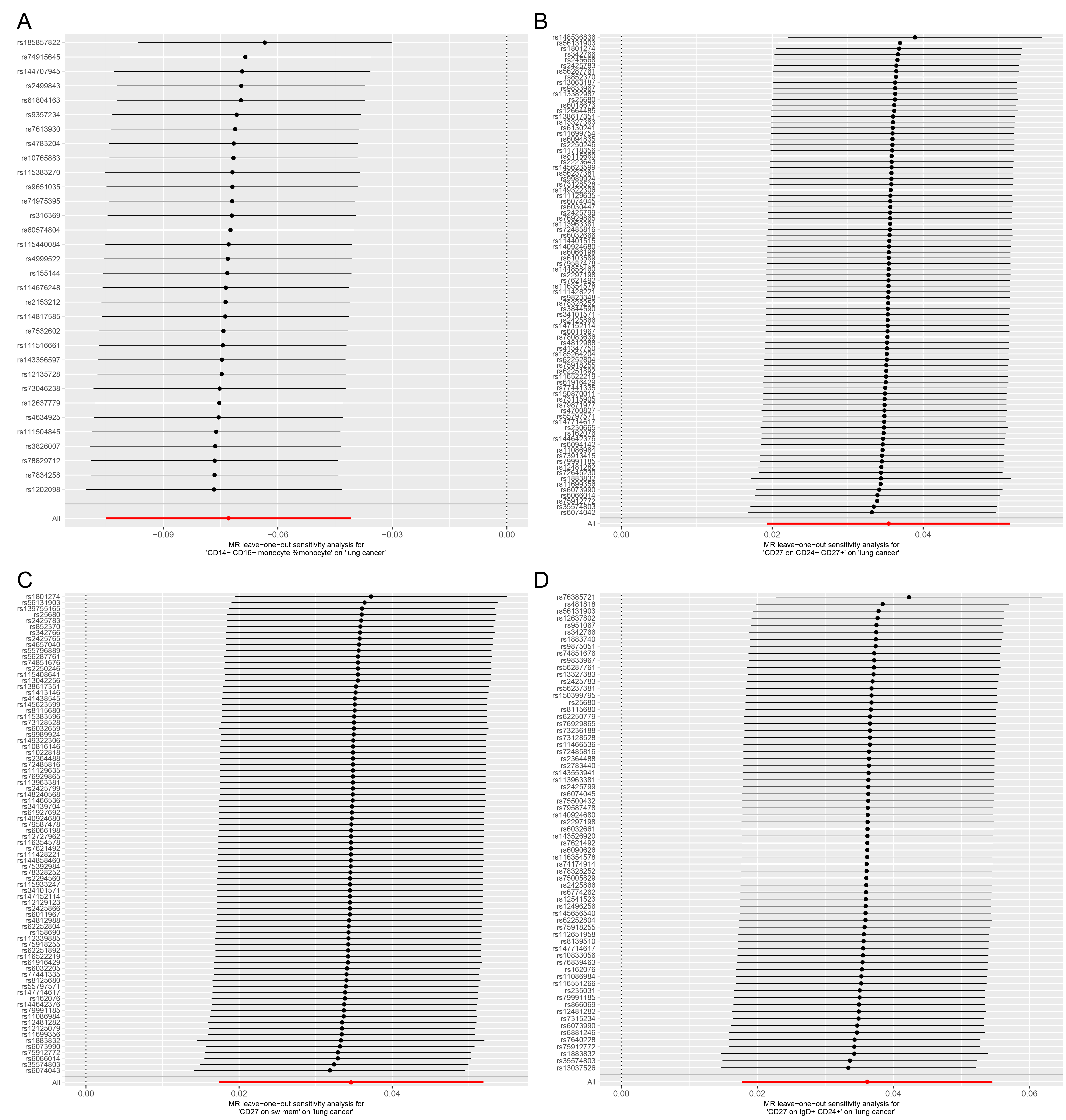
**Figure S3:** Leave-one-out stability tests of the univariable mendelian randomization analyses. Calculate the MR results of the remaining IVs after removing the IVs one by one (A: CD14^-^ CD16^+^ monocyte, B: CD27 on CD24^+^ CD27^+^ B cell, C: CD27 on switched memory B cell, D: CD27 on IgD^+^ CD24^+^ B cell).
